# The better the story, the bigger the serving: narrative transportation increases snacking during screen time in a randomized trial

**DOI:** 10.1186/1479-5868-10-60

**Published:** 2013-05-16

**Authors:** Elizabeth J Lyons, Deborah F Tate, Dianne S Ward

**Affiliations:** 1 Department of Nutrition, Lineberger Comprehensive Cancer Center, The University of North Carolina at Chapel Hill, Chapel Hill, NC, USA; 2 Department of Health Behavior, Department of Nutrition, Lineberger Comprehensive Cancer Center, The University of North Carolina at Chapel Hill, Chapel Hill, NC, USA; 3 Department of Nutrition, The University of North Carolina at Chapel Hill, Chapel Hill, NC, USA; 4 The University of Texas Medical Branch, 301 University Blvd, Galveston, TX 77555-0342, USA

## Abstract

**Background:**

Watching television and playing video games increase energy intake, likely due to distraction from satiety cues. A study comparing one hour of watching TV, playing typical video games, or playing motion-controlled video games found a difference across groups in energy intake, but the reasons for this difference are not clear. As a secondary analysis, we investigated several types of distraction to determine potential psychosocial mechanisms which may account for greater energy intake observed during sedentary screen time as compared to motion-controlled video gaming.

**Methods:**

Feelings of enjoyment, engagement (mental immersion), spatial presence (the feeling of being in the game), and transportation (immersion in a narrative) were investigated in 120 young adults aged 18 – 35 (60 female).

**Results:**

Only narrative transportation was associated with total caloric intake (ρ = .205, P = .025). Transportation was also higher in the TV group than in the gaming groups (P = .002) and higher in males than in females (P = .003). Transportation mediated the relationship between motion-controlled gaming (as compared to TV watching) and square root transformed energy intake (indirect effect = −1.34, 95% confidence interval −3.57, −0.13). No other distraction-related variables were associated with intake.

**Conclusions:**

These results suggest that different forms of distraction may differentially affect eating behavior during screen time, and that narrative appears to be a particularly strong distractor. Future studies should further investigate the effects of narrative on eating behavior.

## Background

Television watching is associated with numerous negative health outcomes, including obesity, metabolic syndrome, and premature death [1,2]. One of the mechanisms by which television watching affects health is increased snacking during television watching periods. Though food advertisements appear to be partially responsible for this affect, multiple studies suggest that TV also increases energy intake due to distraction from satiety cues [3-6]. In fact, a comparison of watching a continuous TV program to watching brief clips from that program found greater intake during the continuous program condition [7]. The authors suggested that increased attentional allocation, or distraction, may have been responsible for this difference. Conceptually distinct types of distraction exist that may differentially affect intake. Though many distraction-related terms are used interchangeably, here we contrast engagement, spatial presence, and narrative transportation as representing different depths of immersion.

Engagement, or mental immersion, is a measure of attentional allocation [8]. As defined by the International Society for Presence Research, engagement occurs when one’s perception is directed towards a technologically mediated world and away from the physical world [9]. When engaged with, for example, a television program, one’s attention is selectively allocated to the program at the expense of one’s environment. Common synonyms for engagement include involvement, immersion, and engrossment.

It is possible to become so engaged in a technologically-mediated world as to feel as if one is physically present in it. This sensation is known as spatial or physical presence [10]. In addition to feelings of engagement in the world, individuals also suspend their disbelief in its physicality. For example, “jump scares” in horror or other media cause startle reflexes, even though viewers intellectually realize that the objects are not real [11]. Thus, spatial presence consists not only of attentional distraction but also a psychological feeling of being in an alternative space.

Narrative transportation, or absorption in a storyline, integrates attentional allocation with imagery and feelings related to a story [12]. When absorbed in a narrative, as opposed to non-narrative media, a loss of self-awareness is combined with mental construction of the narrative reality. Though engagement and presence in a virtual environment may be relatively passive, individuals must actively participate in imagining a storyline. Thus, narrative transportation may produce more profound distraction than engagement or even spatial presence because of the mental effort required to construct the narrative.

Results of a recent study suggested that sedentary screen time, including TV watching and typical video gaming, can produce greater energy intake than playing video games that use motion-based controls, and that this effect is unlikely to be due to lower levels of energy expenditure [13]. Motion-controlled games played with a camera-based or accelerometer controller (such as Microsoft’s Kinect or Nintendo’s Wiimote) require body movement to play and may reduce intake by busying hands, reducing opportunities to eat as much as in conditions with idle hands (e.g., TV watching). These games may also be less distracting than sedentary games and TV watching. There is mixed evidence as to feelings of presence and engagement during motion-controlled video games as compared to typical video games [14-18], and little is known about narrative transportation. Previous studies have compared sedentary gaming to no stimulus [19,20] and to gaming while walking on a treadmill [21], but we are unaware of TV or gaming studies that have measured or compared the effects of different types of distraction.

The purpose of this secondary data analysis was to investigate psychosocial variables measured during a study comparing TV watching, typical video gaming, and motion-controlled video gaming (described above) [13]. The effects on energy intake of several different measures of distraction were studied: transportation, spatial presence, and engagement. We hypothesized that greater distraction would be associated with greater energy intake. We also hypothesized that TV watching and typical sedentary gaming would be more distracting than motion-controlled gaming. The effects of enjoyment and tendency towards immersion were also explored.

## Materials and methods

Details on recruitment, participants, and protocol of the larger study have been provided previously [13]. Briefly, the PRESENCE 2 project recruited 120 young adults aged 18 – 35 (60 female) for a one-hour experimental protocol. Recruitment occurred through a local online mailing list and TV advertisements. Participants were stratified by gender and then randomized into one of three conditions: TV watching, typical video gaming, or motion-controlled video gaming. The TV watching condition consisted of watching commercial-free TV shows using an instant streaming service (Netflix). Participants could choose the shows they wished to watch and change shows at any time. Comedy and drama shows were the most popular. Only two shows included any kind of food-related content (“Ace of Cakes” and “No Reservations”). The typical video gaming condition consisted of playing one or more of 10 possible games on a video game console that used a standard controller (Sony Playstation 3 using a Dualshock controller). The games included a range of genres and ratings and were all rated over 75 out of 100 on a critical ratings aggregator. Playstation 3 was chosen because it offered a wide variety of highly ranked games that were not first-person shooter games. In previous studies, many women have displayed a strong dislike of violent first-person shooters and similar genres [22,23]. The most popular games were Street Fighter IV, LittleBigPlanet, and Call of Duty: Modern Warfare 2. The motion-controlled video game condition consisted of play of one or more of 10 possible games on a Nintendo Wii or Microsoft Xbox360 console (the two Xbox360 games both included their own motion-controller peripheral controller: Dance Dance Revolution: Universe 2 with a dance mat and Rock Band 2 with a drum set controller). To be included, the games had to include at least punching, throwing, or other similar motions. The most popular games were Wii Sports Resort, Rock Band 2, and Punch-Out!! (played using the motion-controlled configuration). Media were shown on a 58” HD TV in a small, dim room. A comfortable chair was available, with sufficient room (~6-8 feet) for standing to play games.

Participants watched TV or played video games for one hour while four types of snacks (chips, baked chips, trail mix, and chocolate candy) and four types of beverage (Coca-Cola, Diet Coke, Mountain Dew, and bottled water) were available. Caloric intake was estimated by weighing food and beverage containers before and after each session using a Tanita food scale (Arlington Heights, IL) and then converting weight to kilocalories based on each snack’s published nutrition data. Baseline and trait psychosocial measures and demographic variables were measured prior to experimentation, and psychosocial outcomes were measured after the one-hour experimentation period.

### Measures

Immersive tendencies were measured using Witmer and Singer’s 16-item Immersive Tendencies Questionnaire [24]. This measure includes items that specifically mention immersion in TV programs, movies, sports, books, games, and storylines, making it an appropriate general measure of tendencies towards all types of distraction. Items include “do you ever become so involved in a television program or book that people have problems getting your attention?” and “have you ever remained apprehensive or fearful long after watching a scary movie?” The focus and involvement subscales were used in this study (a game-specific subscale was excluded). Items from each subscale were summed from 1 – 7 Likert scale responses.

Enjoyment was measured using the interest/enjoyment subscale of the Intrinsic Motivation Inventory, a 7-item measure that has been used in previous gaming studies and that has shown reliability and validity [22,25,26]. Items were altered slightly to be specific to the game or program being discussed, such as “I enjoyed playing the game very much” and “I would describe this program as very interesting.”

Engagement and spatial presence were measured with their respective subscales from the Temple Presence Inventory [27,28]. This measure was created from items used across various presence scales and previously has been used in similar literature [16]. The six-item engagement subscale included questions such as “to what extent did you feel mentally immersed in the experience” and “how completely were your senses engaged?” The four-item spatial presence subscale included questions such as “to what extent did you experience a sense of being there inside the environment you saw/heard?” All items on both subscales used 7-point Likert responses that were averaged for the final score (range: 1 – 7).

Narrative transportation was measured using Green and Brock’s 12-item transportation scale, adapted to reference video games and television shows [29]. Items included “I wanted to learn how the program ended” and “I found myself thinking of ways the game could have turned out differently.” Eleven general items were used with one additional item related to characters (“I had a vivid image of my character” for gaming and “I had a vivid image of the main character” for TV). Items for this measure were summed for a final transportation score (range: 12 – 84).

All scales were found to be reliable (immersive tendencies, 0.81; enjoyment, 0.95; engagement, 0.83; spatial presence, 0.78; transportation, 0.72).

### Data analysis

All analyses were performed using PASW Statistics version 18 (SPSS, Inc., Chicago, IL). Because energy intake was not normally distributed, associations were analyzed using Spearman’s rho. Analyses of covariance were used to determine differences by group assignment and gender for normal variables.

All psychosocial outcome measures were first calculated for each individual game played or program watched. To represent an overall score for the entire hour-long period, scores for all games/programs were averaged for an overall score for each variable. Only those games or programs used for more than five minutes were included in these analyses.

Mediation effects (also known as indirect effects) were tested where associations were found between a psychosocial outcome and square root-transformed energy intake. Preacher and Hayes’ PASW macro “indirect” was used for these analyses. This macro uses bootstrapping to estimate the mediated effect and bias-corrected accelerated 95% confidence interval of the effect [30]. Bootstrapping is a simulation technique that uses the observed sample as representative of a larger population [31]. Using the observed sample, the assumed population is resampled with replacement *k* number of times (here, *k* = 5000). For each simulated sample, the product of paths *a* (from the independent variable to the mediator) and *b* (from the mediator to the dependent variable) are estimated. These products represent a distribution that approximates the sampling distribution of the indirect effect in the population. The 95% confidence interval of the indirect effect provides a method for hypothesis testing such that intervals not including zero represent a significant indirect (mediated) effect. Rather than test the significance of the path coefficients themselves, this technique directly tests the significance of the indirect effect (the a*b product). This technique is considered superior to the causal steps method, which does not directly test a mediated effect, and the Sobel test, which assumes a normal distribution of the indirect effect and has lower statistical power [32].

## Results

### Participant characteristics

Participants were 62% white (n = 74), 17% black (n = 20), 14% Asian (n = 17), 8% other race (n = 8), and 8% of Hispanic ethnicity (n = 9). Most were well-educated (42% having attended some college, 35% having an undergraduate degree, and 16% with a graduate degree). Their mean demographic characteristics were: age 24.07 ± 4.43 years, weight 71.52 ± 14.83 kg, height 170.99 ± 9.77 cm, and BMI 24.41 ± 4.14 kg/m^2^.

Most participants only used one game/program (53 out of 120, 44%) or two (49 out of 120, 41%). The average time spent on the first and second games/programs were 41.49 and 23.84 minutes, respectively.

Immersive tendencies were not associated with energy intake (involvement, ρ = .059, P = .522; focus, ρ = .042, P = .651). Women reported higher tendency toward involvement than men (P = .003). No differences by gender were found for tendency toward focus. No group differences were found for any immersive tendency variables (P > .05).

As reported previously [13], mean (standard deviation) kilocalorie intake was 716 (407) for TV, 747 (540) for video gaming, and 553 (498) for motion-controlled gaming.

### Effects of distraction and enjoyment on intake

Associations between total kcal intake, enjoyment, and psychological indicators of distraction were tested. Narrative transportation showed a significant association with intake (ρ = .205, P = .025), whereas spatial presence (ρ = .061, P = .506), engagement (ρ = .020, P = .832), and enjoyment (ρ = .027, P = .769) did not. Transportation was associated with calories from food (ρ = .183, P = .045) but not from beverages (ρ = .156, P = .089). Enjoyment was associated with transportation (ρ = .507, P < .001) and engagement (ρ = .665, P < .001), but not with presence (ρ .143, P = .119).

### Differences by group and gender

Differences in transportation were found for both gender (F = 9.53, P = .003) and group assignment (F = 6.47, P = .002; Table 1). Males showed higher transportation levels than females (mean difference 4.67, 95% confidence interval 1.67 – 7.66). Compared to TV watching, traditional video games (mean difference 5.16, 95% confidence interval 1.48 – 8.83) and motion-controlled video games (mean difference 6.24, 95% confidence interval 2.57 – 9.91) produced lower levels of transportation.

**Table 1:** Differences by gender and type of screen time, mean (SD)

		**TV watching**		** Video game playing**			** Motion-controlled gaming**			**Total**
	**Male**	**Female**	**Total**	**Male**	**Female**	**Total**	**Male**	**Female**	**Total**	
Transportation	45.72 (6.36)	43.69 (9.51)	44.70* (8.05)	42.01 (9.23)	37.09 (8.01)	39.55 (8.88)	42.00 (8.27)	34.93 (7.98)	38.46 (8.79)	40.91** (8.94)
Presence	1.79 (0.70)	2.09 (1.03)	1.94 (0.89)	2.58 (1.19)	1.86 (0.75)	2.22 (1.05)	2.50 (1.11)	1.98 (0.81)	2.24 (1.00)	2.13 (0.98)
Engagement	4.36 (0.83)	4.42 (0.97)	4.39 (0.90)	4.38 (0.84)	4.12 (0.91)	4.25 (0.87)	4.43 (1.12)	4.42 (1.07)	4.42 (1.09)	4.39 (0.95)
Enjoyment	5.80 (0.56)	5.49 (0.99)	5.64 (0.81)	5.08 (1.06)	5.38 (1.05)	5.23 (1.05)	5.03 (1.04)	5.21 (0.80)	5.12 (0.92)	5.33 (0.95)
Involvement	25.15 (7.46)	29.80 (5.50)	27.48 (6.88)	24.95 (7.04)	26.60 (7.38)	25.78 (7.17)	23.60 (6.02)	28.95 (8.83)	26.28 (7.94)	26.51** (7.32)
Focus	22.55 (4.89)	23.00 (4.28)	22.78 (4.54)	24.10 (4.44)	22.65 (3.70)	23.38 (4.10)	24.20 (3.25)	22.45 (6.44)	23.33 (5.11)	23.16 (4.57)

*Significantly higher than in video game playing or motion-controlled gaming.

**Gender difference, P < .05.

No differences by group or gender were found for engagement (P = .913), spatial presence (P = .074), or enjoyment (P = .099).

### Mediation test

The mediated effect of group assignment on energy intake via narrative transportation was investigated. Dummy variables were used to compare the motion-controlled gaming condition to the TV watching condition, with the sedentary gaming condition as a covariate. The *a*-path from group assignment to transportation was negative (b = −6.24, SE = 1.92), and the *b*-path from transportation to intake was positive (b = 0.22, SE = 0.11). The estimated indirect effect was −1.34, with a bias-corrected and accelerated 95% confidence interval ranging from −3.57 to −0.13, indicating statistical significance. We repeated the analysis with the sedentary gaming condition as the independent variable (and the TV condition as a covariate) to determine whether a mediated effect also existed when comparing motion-controlled gaming to typical sedentary gaming. This analysis did not produce a significant mediated effect (−0.21, 95% confidence interval −1.46, 0.59).

## Discussion

Only one of the three types of distraction measured was associated with energy intake during screen time. Narrative transportation showed a small positive association with energy intake. Engagement and spatial presence were not associated with intake, nor were feelings of enjoyment. Transportation was found to be higher in men and in those watching TV as compared to women and those playing either type of video game. Narrative transportation mediated the effect of group assignment on energy intake, but only when comparing TV watching to motion-controlled gaming. Thus, greater narrative transportation in the TV watching condition, as compared to the motion-controlled gaming condition, contributed to greater energy intake.

Several previous studies have compared the impacts of different forms of media on energy intake. No difference was found between TV watching and listening to a detective story [5], but TV watching has produced greater energy intake when compared to listening to a symphony [33]. When a continuous TV program was compared to loops of 1.5-minute portions of the same show, the continuous program produced greater energy intake [7]. Narrative-based media may be particularly distracting and thus lead to greater energy intake than non-narrative media. Health promotion efforts currently use narrative to model healthy behaviors [34] and to persuade individuals to change their behaviors [35,36]. Narrative may be useful for increasing “self” presence or character identification, which can promote feelings of social support and belonging [37]. However, the potential of highly transporting narratives to increase positive energy balance should be considered prior to their use in future studies.

### Limitations

The data analyzed here represented averages of multiple programs or games used over a one-hour period. It is unknown why participants chose to stop one program or game and begin another. The nature of the study design, with a primary intent on comparing three types of screen time while allowing participants to view/play their preferred titles within those types, precludes precise study of individual titles. Though only two of the TV shows watched were related to food, other cues may have influenced participant desire to eat. Gender differences may have resulted from differences in the content chosen by each gender; further study of the effects of content on distraction is necessary to better understand this finding. We have provided frequencies for all television shows watched and video games played by gender in an Additional file 1. These data suggest that gender differences in video game choice may have existed and thus contributed to differences in distraction. These results are preliminary and intended to provide a basis for more rigorous future investigations of different types of distraction.

The types of food and beverages available may also have influenced intake. It appears that much of the kilocalorie intake across all groups came from trail mix (see Additional file 2). The reasons for this differential intake of one food are unclear but may be related to a perception that trail mix was healthier than the other options, which was expressed by several participants (data not reported).

The transportation scale was not developed for use with television or video game narratives, and the immersive tendencies questionnaire may not adequately measure the tendency to be transported specifically by a narrative, as it is general to all types of presence. Self-reported distraction, reported after the experimental period, is not an optimal method of measurement. Future studies could improve on the measures presented here by including measures of attentional focus and distraction types during the experimental period. Further validation of self-report measures would also strengthen future experiments.

## Conclusions

Narrative transportation was lower for motion-controlled video games, leading to lower energy intake as compared to TV watching. Story-related immersion appears to be uniquely effective as a distraction, producing an effect when engagement and spatial presence did not. Future research is necessary to further investigate the potential of highly involving narratives to distract from bodily stimuli, which could have positive implications across a number of public health fields. Future research should also determine methods of ameliorating the potential negative effects of narrative transportation due to increased energy intake.

## Competing interests

The authors declare that they have no competing interests.

## Authors’ contributions

EL, DFT, and DSW designed the study. EL collected and analyzed the data and wrote the manuscript. DFT and DSW edited the manuscript. All authors read and approved the final manuscript.

## Supplementary Material

Additional file 1 Television shows watched and video games played by gender. Click here for file

Additional file 2 Food and beverage intake. Click here for file
