# Supplementary material for: The better the story, the bigger the serving: narrative transportation increases snacking during screen time in a randomized trial
Source: Int J Behav Nutr Phys Act. 2013 May 16;10:60. doi: 10.1186/1479-5868-10-60 (PMC3660271; doi:10.1186/1479-5868-10-60)
Supplement: Additional file 1 — Television shows watched and video games played by gender. [file 1479-5868-10-60-S1.doc]

**Additional file 1. Television shows watched and video games played by gender**

General notes:

- Frequencies represent times watched or played, not necessarily individuals. Though most participants watched/played 1-2 media per session, some sampled from a number of different games/shows.

***Section 1. Television shows***

Notes:

- Television shows were divided into genres based upon the broadest genre description provided by Netflix (e.g., drama as a broader category than crime)
  - TV Action & Adventure was included in the drama category
- We created a separate “animated” genre combining cartoons and “Kids’ TV”

Table 1.1. Frequencies for animated television shows watched ≥ 5 minutes by gender

| Show name | Male | Female |
| --- | --- | --- |
| Avatar: The Last Airbender | 1 | 0 |
| Family Guy | 1 | 1 |
| Futurama | 0 | 1 |
| Ren and Stimpy | 1 | 0 |
| South Park | 1 | 0 |

Table 1.2. Frequencies for comedy television shows watched ≥ 5 minutes by gender

| Show name | Male | Female |
| --- | --- | --- |
| 30 Rock | 2 | 3 |
| Arrested Development | 1 | 1 |
| Chappelle’s Show | 2 | 0 |
| Fry & Laurie | 0 | 1 |
| The Guild | 1 | 0 |
| The IT Crowd | 1 | 0 |
| The Office | 2 | 3 |
| Parks and Recreation | 1 | 0 |
| Party Down | 0 | 1 |
| Psych | 1 | 0 |
| Saturday Night Live | 1 | 0 |
| The State | 0 | 1 |
| Stella | 0 | 1 |
| Strangers With Candy | 1 | 0 |
| The Tick | 1 | 0 |

Table 1.3. Frequencies for drama television shows watched ≥ 5 minutes by gender

| Show name | Male | Female |
| --- | --- | --- |
| 24 | 0 | 2 |
| Buffy the Vampire Slayer | 0 | 1 |
| Conan | 1 | 0 |
| Dexter | 1 | 2 |
| Farscape | 1 | 0 |
| Grey’s Anatomy | 0 | 1 |
| The Tudors | 0 | 1 |
| Weeds | 2 | 1 |

Table 1.4. Frequencies for documentary television shows watched ≥ 5 minutes by gender

| Show name | Male | Female |
| --- | --- | --- |
| Appalachian Trail | 1 | 0 |
| The Blue Planet | 0 | 1 |
| The Dark Ages | 0 | 1 |
| Empires of Industry | 1 | 0 |
| Everest | 0 | 1 |
| Life of Mammals | 2 | 0 |
| The Universe | 2 | 0 |
| Volcanoes | 0 | 1 |

Table 1.5. Frequencies for reality television shows watched ≥ 5 minutes by gender

| Show name | Male | Female |
| --- | --- | --- |
| Cake Boss | 0 | 1 |
| Flavor of Love | 0 | 1 |
| Hoarders | 0 | 1 |
| Intervention | 1 | 0 |
| Jon & Kate Plus 8 | 1 | 0 |
| Mythbusters | 0 | 1 |
| No Reservations | 0 | 1 |
| Say Yes to the Dress | 0 | 1 |

Table 1.6. Frequencies for television shows watched ≥ 5 minutes by genre and gender

| Genre | Male | Female |
| --- | --- | --- |
| Animated | 4 | 2 |
| Comedy | 14 | 11 |
| Drama | 5 | 8 |
| Documentary | 6 | 4 |
| Reality | 2 | 6 |

***Section 2. Video games***

Notes:

- Genre definitions vary widely depending on the source of the definition and assignment. Horror, platform, role-playing, and shooter games all could be considered sub-sets of action games. Similarly, the two games assigned to “action” here could easily be placed in more specific sub-genres of their own, such as Action-Adventure and Fighting. We chose to use this combination of genre categories and to assign the games in this manner to provide the most insight into gender differences by specific sub-genres of game.

Table 2.1. Frequencies for video games played ≥ 5 minutes by gender

| Game name | Male | Female |
| --- | --- | --- |
| 3D Dot Game Heroes | 0 | 2 |
| Assassin’s Creed II | 4 | 2 |
| Call of Duty: Modern Warfare 2 | 10 | 0 |
| Dead Rising 2 | 6 | 2 |
| Dead Space | 3 | 0 |
| Final Fantasy XIII | 2 | 4 |
| Little Big Planet | 3 | 10 |
| Ratchet & Clank | 1 | 3 |
| Red Dead Redemption | 5 | 0 |
| Street Fighter IV | 6 | 7 |

Table 2.2. Frequencies for video games by assigned genre

| Genre | Game name | Male | Female |
| --- | --- | --- | --- |
| Action | Assassin’s Creed II | 4 | 2 |
|  | Street Fighter IV | 6 | 7 |
| Horror | Dead Rising 2 | 6 | 2 |
|  | Dead Space | 3 | 0 |
| Platform | Little Big Planet | 3 | 10 |
|  | Ratchet & Clank Future | 1 | 3 |
| Role playing game | 3D Dot Game Heroes | 0 | 2 |
|  | Final Fantasy XIII | 2 | 4 |
| Shooter | Call of Duty: Modern Warfare 2 | 10 | 0 |
|  | Red Dead Redemption | 5 | 0 |

***Section 3. Motion-controlled video games***

Notes:

- Assigning genres to motion-controlled games is quite difficult, particularly as these games were chosen based upon the movement they required during play-testing rather than their content per se. We have attempted to provide reasonable genre assignments to these games to best illustrate potential gender differences by game content, but these assignments should be taken as preliminary.

Table 3.1. Frequencies for video games played ≥ 5 minutes by gender

| Game name | Male | Female |
| --- | --- | --- |
| Boom Blox Bash | 0 | 5 |
| Dance Dance Revolution | 0 | 7 |
| NHL Slapshot | 4 | 0 |
| Punch-Out!! | 8 | 2 |
| Rayman Raving Rabbids | 4 | 4 |
| Rock Band 2 | 7 | 5 |
| Wario Ware: Smooth Moves | 1 | 1 |
| We Cheer | 1 | 1 |
| Wii Fit Plus | 1 | 6 |
| Wii Sports Resort | 13 | 7 |

Table 3.2. Frequencies for video games by assigned genre

| Genre | Game name | Male | Female |
| --- | --- | --- | --- |
| Dance games | Dance Dance Revolution | 0 | 7 |
|  | We Cheer | 1 | 1 |
| Party games | Boom Blox Bash | 0 | 5 |
|  | Rayman Raving Rabbids | 4 | 4 |
|  | Wario Ware: Smooth Moves | 1 | 1 |
| Band simulation games | Rock Band 2 | 7 | 5 |
| Sports games | NHL Slapshot | 4 | 0 |
|  | Punch-Out!! | 8 | 2 |
| “Wii” branded minigame collections | Wii Fit Plus | 1 | 6 |
|  | Wii Sports Resort | 13 | 7 |
