# Supplementary material for: The better the story, the bigger the serving: narrative transportation increases snacking during screen time in a randomized trial
Source: Int J Behav Nutr Phys Act. 2013 May 16;10:60. doi: 10.1186/1479-5868-10-60 (PMC3660271; doi:10.1186/1479-5868-10-60)
Supplement: Additional file 2 — Food and beverage intake. [file 1479-5868-10-60-S2.doc]

**Additional file 2. Food and beverage intake**

Notes:

- Serving size was taken from the packaging for each food/beverage
- Kilocalories were calculated from grams using published nutrition information for each food/beverage

Table 1. Energy intake for each type of food and beverage, median (interquartile range)

|  | Grams | Servings | Kilocalories |
| --- | --- | --- | --- |
| Baked Lay’s | 0.0 (16.0) | 0.0 (0.6) | 0.0 (68.6) |
| Doritos | 14.5 (45.0) | 0.5 (1.6) | 77.7 (228.8) |
| M & M’s | 22.0 (57.8) | 0.5 (1.4) | 110.0 (288.8) |
| Trail Mix | 32.5 (91.0) | 1.1 (3.0) | 162.6 (68.6) |
| Coca-Cola | 0.0 (84.0) | 0.0 (0.2) | 0.0 (31.9) |
| Diet Coke | 0.0 (0.0) | 0.0 (0.0) | -- |
| Mountain Dew | 0.0 (0.0) | 0.0 (0.0) | 0.0 (0.0) |
| Water | 195.5 (504.8) | 0.6 (1.5) | -- |

Table 2. Servings by group and gender, median (interquartile range)

|  | Television | | Video games | | Motion controlled video games | |
| --- | --- | --- | --- | --- | --- | --- |
|  | Male | Female | Male | Female | Male | Female |
| Baked Lay’s | 0.0 (0.8) | 0.0 (0.4) | 0.5 (0.8) | 0.0 (0.5) | 0.2 (1.0) | 0.0 (0.4) |
| Doritos | 1.0 (2.4) | 0.5 (1.6) | 1.0 (1.7) | 0.3 (1.7) | 0.1 (1.2) | 0.0 (0.8) |
| M & M’s | 0.6 (1.2) | 0.8 (1.4) | 1.5 (2.1) | 0.6 (1.1) | 0.5 (1.4) | 0.0 (0.4) |
| Trail Mix | 1.5 (3.1) | 1.6 (2.9) | 2.0 (5.2) | 0.2 (1.2) | 2.0 (4.4) | 0.0 (1.6) |
| Coca-Cola | 0.0 (1.0) | 0.0 (0.7) | 0.0 (0.8) | 0.0 (0.0) | 0.0 (0.7) | 0.0 (0.0) |
| Diet Coke | 0.0 (0.0) | 0.0 (0.0) | 0.0 (0.0) | 0.0 (0.0) | 0.0 (0.0) | 0.0 (0.0) |
| Mountain Dew | 0.0 (0.0) | 0.0 (0.0) | 0.0 (1.0) | 0.0 (0.0) | 0.0 (0.0) | 0.0 (0.0) |
| Water | 1.5 (1.5) | 0.7 (1.5) | 0.0 (0.9) | 0.7 (1.3) | 0.3 (1.1) | 0.3 (1.4) |
